# Prognostic significance of preoperative MRI findings in young patients with breast cancer

**DOI:** 10.1038/s41598-019-39629-w

**Published:** 2019-02-28

**Authors:** Almir G. V. Bitencourt, Deise S. G. Eugênio, Juliana A. Souza, Juliana O. Souza, Fabiana B. A. Makdissi, Elvira F. Marques, Rubens Chojniak

**Affiliations:** 1 A.C. Camargo Cancer Center, Imaging Department, São Paulo, 01509-010 Brazil; 2 A.C. Camargo Cancer Center, Mastology Department, São Paulo, 01509-010 Brazil

## Abstract

The objective is to evaluate the prognostic value of preoperative magnetic resonance imaging (MRI) findings in breast cancer patients aged less than 40 years. This retrospective, single-center study evaluated 92 women aged <40 years who received a diagnosis of invasive breast carcinoma between 2008 and 2012. These patients underwent a breast MRI before treatment and follow-up at the same institution. Kaplan-Meier survival curves were used to analyze overall survival, with the log-rank test used to compare different groups. Cox regression analysis was used to estimate hazard ratios (HRs) with 95% confidence interval (95% CI) values. The mean age of the patients was 34 years (range: 25–39 years) and the mean tumor size was 3.9 cm in maximal dimension (range: 0.7–10.5 cm). Recurrence was observed in 21 (22.8%) patients and 15 (16.3%) patients did not survive during a mean follow-up period of 5.4 ± 1.9 years. MRI findings associated with worse overall survival included tumor size >5 cm (HR:5.404; 95% CI:1.922–15.198; p = 0.017), presence of non-mass enhancement (HR:3.730; 95% CI:1.274–10.922; p = 0.016) and multifocal tumor (HR:3.618; 95% CI:1.151–11.369; p = 0.028). Inconclusion, MRI findings that are suggestive of more extensive disease were associated with worse overall survival in young breast cancer patients.

## Introduction

Less than 7% of all breast cancer cases in the United States are diagnosed in women younger than 40 years of age^1^. However, young age has been identified as an independent negative prognostic factor in patients with breast cancer^2^. Moreover, compared with older patients, breast cancer patients aged less than 40 years exhibit increased frequency of hormone receptor-negative tumors, aggressive tumor characteristics, advanced TNM staging, and poorer clinical outcome^3–5^.

Magnetic resonance imaging (MRI) is frequently used as a complementary method for extent of disease evaluation in high-risk patients, in patients with dense breast tissue, and in patients aged less than 40 years^6^. MRI has exhibited greater accuracy compared with mammography and ultrasound in assessing tumor extent and detecting multifocal and multicentric disease, both of which play an important role in the locoregional staging of breast cancer and its treatment planning^7^. Preoperative breast MRI has been shown to affect surgical management in a significant proportion of younger women diagnosed with breast cancer^8–10^. However, the prognostic importance of preoperative MRI features in this particular population has not previously been evaluated.

Therefore, the objective of this study was to evaluate the prognostic value of preoperative MRI findings in breast cancer patients aged less than 40 years.

## Methods

This descriptive, retrospective, single-center study evaluated 120 patients who were diagnosed with breast cancer before the age of 40 years between November 2008 and August 2012. The study was approved by the institutional ethics committee and all methods were carried out in accordance with relevant guidelines and regulations. Due to the retrospective design of the study, the informed consent was waived. Among those 120 patients, 28 patients were excluded due to absence of a breast MRI before treatment (n = 15), a diagnosis of low-grade ductal carcinoma *in situ* (DCIS) without MRI findings (n = 2), metastatic disease present at diagnosis (n = 8), and insufficient follow-up in the institution (<12 months) (n = 3). Thus, a total of 92 patients were included in the final analysis.

MRI studies were conducted with a high-field system (1.5 Tesla, GE Medical Systems, Milwaukee, WI, USA) with a dedicated 8-channel breast coil and use of intravenous paramagnetic contrast. Each examination consisted of images taken before and after the use of paramagnetic contrast (gadopentetate dimeglumine; Gd-DTPA) in a 20 ml dose and speed infusion of 3 ml/s. Before contrast administration, a T1 gradient-echo phase, three dimensional (3D) in the axial plane with 2.5-mm-thick slices and a fat-saturated STIR sequence in the sagittal plane of both the breasts, with 4-mm-thick slices are acquired. Five gradient-echo phases in T1, 3D, in the axial plane are obtained using fat suppression for dynamic examination. The first phase is obtained before contrast injection, the second after 20 s of contrast injection and the other sequentially in the following minutes with a temporal resolution of 60–90 s. Post-processing images subtracting the pre-contrast of post-contrast images are obtained from these dynamic images for better viewing of the highlight areas. The last sequence consists of a sagittal T1-weighted 3D gradient-echo pulse sequence, with slice thickness of 1 mm and fat signal suppression. Images were reviewed by one radiologist with 8 years’ experience in breast imaging. Radiological descriptors were used from the 5th Edition of the American College of Radiology - Breast Imaging Reporting and Data System (ACR-BIRADS) lexicon. Main tumor size was measured on the largest diameter in the post-contrast phase in which the lesion was more evident. Multifocality and multicentricity were defined as the presence of additional malignant lesions in the same quadrant versus different quadrants, respectively, of a primary tumor in the same breast.

Histopathology was performed following surgical resection and these data were available for most of the cases examined. For the patients who submitted to neoadjuvant chemotherapy, data from ultrasound-guided percutaneous core needle biopsy before the treatment were used. Tumor histological types were reported according to the World Health Organization (WHO) classification of tumors. Immunohistochemical data was used to classify breast carcinomas into four molecular subtypes: luminal A (expression of estrogen/progesterone receptors and low proliferation index); luminal B (positive for estrogen and/or progestin receptors with Her-2 overexpression or high proliferation index); Her-2 (negative hormone receptor expression with HER-2 overexpression); and triple-negative (both hormone receptor and Her-2 negative). Pathologic diagnoses, including molecular subtype, were obtained from the patients’ medical reports. A cut-off value of 15% was routinely used for Ki-67 expression to differentiate low versus high proliferation indices.

Statistical analyses were performed by using SPSS for Windows, version 20.0 (SPSS Inc., Chicago, IL, USA). Frequency analysis was performed to characterize the samples. The following tests were used to compare variables: Chi-square test was applied when both variables were categorical, while Student’s *t*-test and the Mann-Whitney test were applied when one of the variables was continuous with and without normal distribution, respectively. Patient age was categorized into two groups: <30 years and >30 years. Tumor size was classified into three groups according to TNM classification: <2.0 cm (T1); 2.1–5.0 cm (T2); and >5.0 cm (T3). Kaplan-Meier survival curves were used to analyze overall survival, with the log-rank test used to compare different groups. Cox regression was used to estimate hazard ratios (HRs) with 95% confidence interval (95% CI) values. The level of significance was set at 5%.

## Results

The mean age of our cohort (n = 92) was 34 ± 3.7 years (range: 25–39 years), with 20 (21.7%) patients aged less than 30 years. Most of the patients were diagnosed with invasive carcinoma of no special type (75.0%) and the most common molecular subtypes were luminal B (n = 41; 50.6%) and triple-negative (n = 19; 23.5%). Table 1 summarizes the pathological data examined. Tumor size was evaluated in 70 patients (22 patients who underwent neoadjuvant chemotherapy were not included in this analysis). The mean tumor size was 4.8 ± 3.5 cm (range: 0.2–12.0 cm), including 26 (28.3%) tumors that were classified as T1, 33 (35.5%) tumors that were classified as T2, and 11 (12.0%) tumors that were classified as T3. Multifocality was observed in 12 (13.0%) patients and multicentricity was observed in 11 (12.0%) patients.

**Table 1:** Pathological data for our cohort of young breast cancer patients (n = 92).

Pathological Finding	N (%)
**Histological type** ^*^	
NST	69 (77.5)
Lobular invasive carcinoma	6 (6.7)
Medullar carcinoma	3 (3.4)
Other special types	11 (12.4)
**Molecular subtype** ^**^**^	
Luminal A	11 (13.5)
Luminal B	41 (50.6)
HER-2	10 (12.3)
Triple-negative	19 (23.4)
**Associated DCIS** ^**#**^	
Present	59 (68.6)
Absent	27 (31.3)
**Nuclear grade** ^**#**^	
I	5 (5.8)
II	18 (20.9)
III	63 (73.3)

Missing data points: *n = 3; ^^^n = 11; ^#^n = 6.

NST: no special type; DCIS, ductal carcinoma *in situ*.

The MRI findings showed that most of the lesions examined were classified as mass (n = 58; 63.0%), while 14 (15.2%) lesions were classified as non-mass enhancement (NME) and 20 (21.7%) lesions were classified as both mass and NME. Table 2 summarizes the MRI findings. Mean tumor size as measured by MRI was 3.9 ± 2.8 cm (range: 0.7–10.5 cm), including 26 (28.3%) tumors which were classified as T1, 43 (46.7%) tumors which were classified as T2, and 23 (25.0%) tumors which were classified as T3. Multifocality was observed in 41 (44.9%) patients and multicentricity was observed in 31 (33.7%) patients.

**Table 2:** MRI findings in young breast cancer patients (n = 92).

MRI Finding	N (%)
**Mass (n = 78)**	
**Shape**	
Round	3 (3.8)
Oval	38 (48.7)
Irregular	37(47.4)
Margin*	
Circumscribed	10 (13.5)
Irregular	45 (60.8)
Spiculated	19 (25.6)
**Kinetic curve assessment** ^**^**^	
Persistent	3 (5.8)
Plateau	20 (38.4)
Washout	29 (55.8)
**Non-mass enhancement (n = 34)**	
**Distribution**	
Segmental	17 (50.0)
Regional	5 (14.7)
Multiple regions	5 (14.7)
Focal	3 (8.8)
Linear	3 (8.8)
Diffuse	1 (2.9)
Internal enhancement pattern*	
Homogeneous	7 (23.3)
Heterogeneous	18 (60.0)
Clumped	4 (13.3)
Clustered rings	1 (3.3)

Missing data points: *n = 4; ^^^n = 26.

Fifteen patients (16.3%) did not survive the mean follow-up period of 5.4 ± 1.9 years. In each case, the cause of death was disease-related. Recurrence was observed in 21 (22.8%) patients, including 1 patient who presented with locoregional recurrence in the chest wall and 20 patients who presented with distant metastasis. Among the latter, the most common metastasis sites included: bone (n = 15), liver (n = 11), lung (n = 10), brain (n = 7), lymph nodes (n = 5), kidney (n = 1), and ovary (n = 1). Recurrence was more common in patients with larger primary tumors and NME at preoperative MRI, as well as in patients with larger tumors and metastasis in axillary lymph nodes (Table 3).

**Table 3:** Correlation between recurrence and clinicopathological factors and MRI findings in young breast cancer patients (n = 92).

Clinicopathological Factor	Recurrence, N (%)		p-value
	No	Yes	
**Age, years**			0.50
≤30	16 (80.0)	4 (20.0)	
>30	55 (76.4)	17 (23.6)	
**Lesion Type**			**0.04**
Mass only	49 (84.5)	9 (15.5)	
Non-mass enhancement	22 (64.7)	12 (35.3)	
Multifocality at MRI*			0.07
No	41 (83.7)	8 (16.3)	
Yes	28 (68.3)	13 (31.7)	
**Multicentricity at MRI** ^**^**^			0.38
No	46 (79.3)	12 (20.7)	
Yes	23 (74.2)	8 (25.8)	
**Tumor size at MRI**			**0.01**
T1	25 (96.2)	1 (3.8)	
T2	31 (72.1)	12 (27.9)	
T3	15 (65.2)	8 (34.8)	
**Molecular Subtype** ^**#**^			0.72
Luminal A	8 (72.7)	3 (27.3)	
Luminal B	29 (70.7)	12 (29.3)	
Her2	7 (70.0)	3 (30.0)	
Triple-negative	16 (84.2)	3 (15.8)	
**Tumor size at Pathology** ^**+**^			**0.01**
T1	26 (100)	0 (0)	
T2	24 (72.7)	9 (27.3)	
T3	8 (72.7)	3 (27.3)	
**Associated DCIS** ^**α**^			0,28
No	22 (81.5)	5 (18.5)	
Yes	43 (72.9)	16 (27.1)	
**Multifocality at Pathology** ^**β**^			0.40
No	40 (74.1)	14 (25.9)	
Yes	10 (83.3)	2 (16.7)	
**Multicentricity at Pathology** ^**χ**^			0.50
No	40 (75.5)	13 (24.5)	
Yes	9 (81.8)	2 (18.2)	
**Axillary Metastasis** ^**#**^			**<0.01**
No	45 (93.8)	3 (6.3)	
Yes	18 (54.5)	15 (45.5)	

Missing data points: *n = 2; ^^^n = 3; ^#^n = 11; ^+^n = 22; ^α^n = 6; ^β^n = 26; ^χ^n = 28.

**Figure 1:**
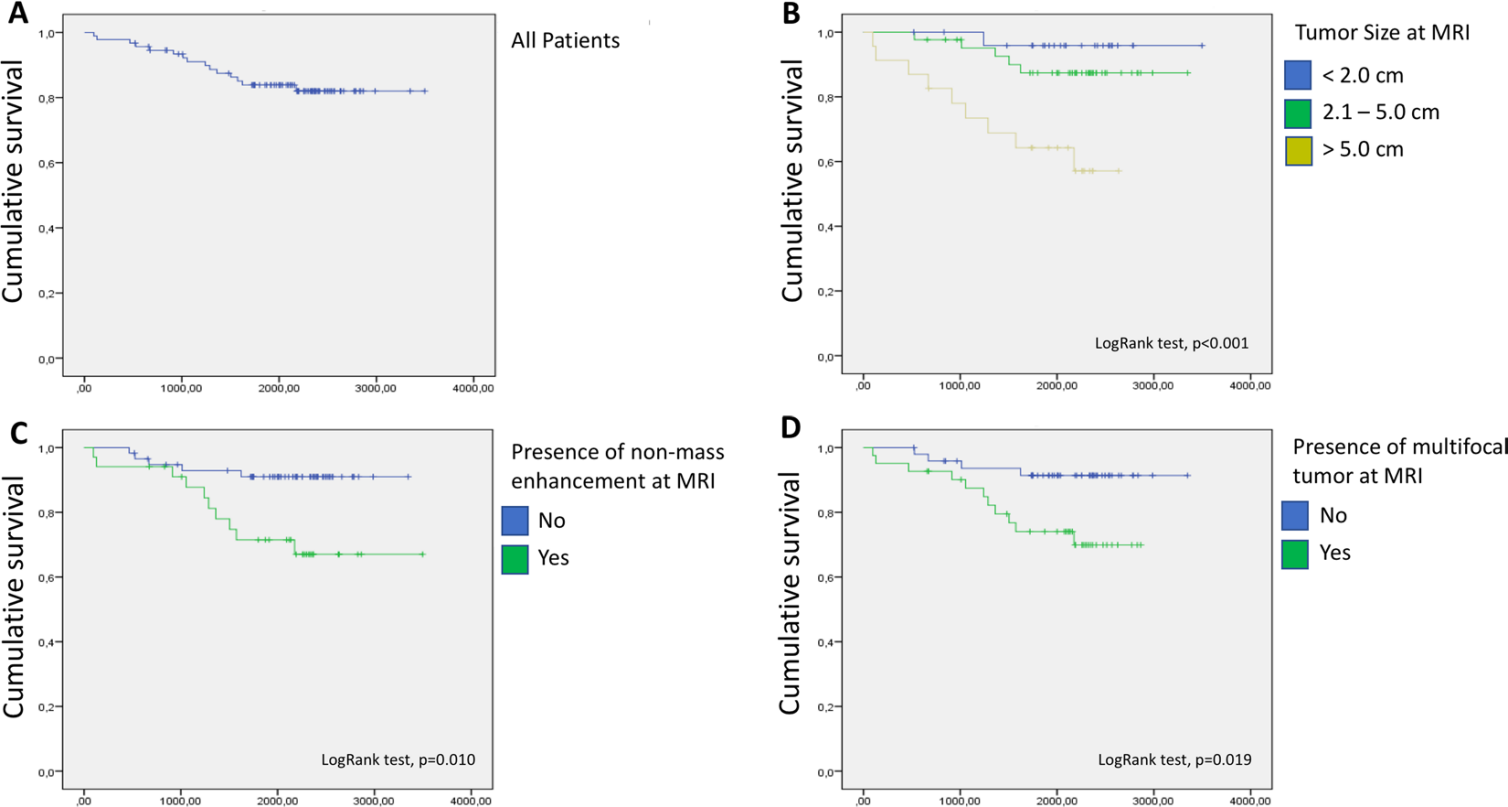
Kaplan-Meier survival curve analyses of breast cancer patients younger than 40 years of age. Survival curves of the overall cohort (**A**) and according to: tumor size as indicated (**B**), presence or absence of NME (**C**), and presence or absence of a multifocal tumor (**D**) at preoperative MRI.

Kaplan-Meier survival curve analysis was performed (Fig. 1) and MRI findings that were associated with worse overall survival included: tumor size >5 cm (log-rank test; p = 0.001), presence of NME (log-rank test; p = 0.010), and multifocality (log-rank test; p = 0.019). Pathological findings that were associated with worse survival included tumor size >5 cm (log-rank test; p = 0.003) and presence of axillary lymph node metastasis (log-rank test; p = 0.005). Table 4 describes the HR, 95% CI, and p-values that were measured by Cox regression analysis for these variables. There was no statistically significant association observed between overall survival and age of diagnosis (log-rank test; p = 0.784), multicentricity at MRI (log-rank test; p = 0.472), molecular subtypes (log-rank test; p = 0.779), multifocality (log-rank test; p = 0.906), multicentricity (log-rank test; p = 0.939), or associated intraductal component (log-rank test; p = 0.833) at pathology.

**Table 4:** Cox regression analysis of overall survival according to clinicopathological and MRI findings in young breast cancer patients (n = 92).

Findings	HR	95% CI	p-value
Non-mass enhancement at MRI	3.730	1.274–10.922	0.016
Multifocality at MRI	3.618	1.151–11.369	0.028
T3 tumor size at MRI	5.404	1.922–15.198	0.001
T3 tumor size at pathology	7.991	1.783–35.821	0.007
Axillary lymph node metastasis	5.212	1.433–18.950	0.012

## Discussion

Prognostic imaging biomarkers are critically needed to improve and optimize breast cancer care. However, only a few studies have evaluated prognosis in relation to breast cancer features detected with MRI. To our knowledge, this is the first study to evaluate the prognostic value of MRI findings in young women with breast cancer.

The recurrence and mortality rates in our cohort were 22.8% and 16.3%, respectively, during a mean follow-up period of 5.4 years. When Larson *et al*. evaluated 322 breast cancer patients aged 40 years or younger at a single institution between 2006 and 2013, the recurrence and mortality rates reported were 17.4% and 8%, respectively, during a mean follow-up period of 4.2 years^11^. Similar to the present results, Larson *et al*. also found that metastatic recurrence (15.2%) was more common than locoregional recurrence (5.6%), and the former occurred more frequently within the first two years after surgery^11^.

The only pathological findings found to be related to poor prognosis in the present study were tumor size >5 cm and node positivity. In contrast, there was no statistically significant difference in patient survival based on molecular subtype. The latter observations are consistent with those of previous studies^11,12^, thereby suggesting that traditional pathological features have a higher predictive power than tumor biology in young patients with breast cancer. In a study conducted by Kim *et al*.^5^, young age was identified as a poor prognostic factor in patients with luminal and Her-2 positive tumors, but not for triple-negative tumors^5^. In the present study, patients with luminal B and Her-2 molecular subtypes of breast cancer exhibited higher recurrence rates and worse survival compared to patients with triple-negative tumors, although the differences were not statistically significant.

Our results show that tumor size, NME, and presence of a multifocal tumor at preoperative MRI are associated with worse prognosis in young breast cancer patients. These findings are usually associated with the presence of a larger and more extensive tumor that is associated with extensive intraductal carcinoma. Lacconi *et al*. (2016) reported that unsuspected additional cancers that are detected only by MRI are often clinically relevant^13^. However, we did not find a statistically significant association between recurrence or survival and the presence of multifocality or an associated intraductal component at pathological analysis after surgery. There are two probable causes for this discrepancy between MRI and pathology evaluations. First, small additional lesions detected with MRI can be difficult to identify by pathological analysis of surgical specimens. Second, some patients underwent neoadjuvant chemotherapy, and this resulted in the treatment of additional foci and associated intraductal carcinoma before the final pathological analysis was performed.

The prognostic value of MRI findings in breast cancer patients of all ages has been shown to vary. For example, Jiang *et al*.^14^ evaluated 88 patients with invasive breast carcinoma to investigate a possible correlation between NME and prognostic factors. NME was more commonly observed in women younger than 50 years, although it was associated with a larger tumor size and was related to low histologic grade and the presence of DCIS^14^. It is important to differentiate NME from background parenchymal enhancement (BPE), which represent the normal enhancement pattern of the normal breast. However, BPE may manifest as a more focal or asymmetric distribution, which can be hard to distinguish from NME^15^. Besides that, some authors have suggested that moderate or marked BPE may also be associated to worse prognosis in patients with breast cancer^16,17^.

Song *et al*.^14^ performed a case-control study to investigate whether breast MRI features were associated with distant metastasis-free survival after controlling for clinicopathologic variables known to be risk factors for distant metastasis^18^. No statistically significant association between the presence of NME or multifocality with distant metastasis-free survival was observed. Furthermore, the only MRI features that were associated with poor prognosis were rim enhancement and peritumoral edema, both of which are more frequently related to triple-negative breast cancers^13^.

There were limitations associated with this retrospective study that should be considered in the analysis of its results. First, we did not match treatment type or presence of genetic mutations, and this could have affected survival outcome. Second, there are MRI factors that have previously been associated with a poor prognosis and these were not evaluated in the present study [e.g., BPE^16,17^, peritumoral edema^18,19^, and kinetic features^20^. Lastly, because of the relatively small number of patients and events at follow-up, the study may be not adequately powered to detect significant differences in survival based on molecular subtype or to perform proper multivariate analysis.

In conclusion, MRI findings that are suggestive of more extensive disease, including larger tumor size, NME, and multifocal tumors, were found to be associated with worse overall survival in our cohort of younger breast cancer patients, irrespective of pathological analysis after surgery. However, further studies are needed to confirm these factors as relevant prognostic biomarkers that may help guide the treatment of breast cancer in younger patients.
